# The Effect of Home- and Community-Based Services on Activity Engagement

**DOI:** 10.1093/geroni/igab046.3054

**Published:** 2021-12-17

**Authors:** Peter Sun

**Affiliations:** Washington University in St. Louis, St. Louis, Missouri, United States

## Abstract

This study examined the association between home- and community-based services (HCBS) and social, cognitive, and physical engagement among community-dwelling older adults in the U.S. Data were drawn from the 2012 Health and Retirement Study (HRS). The sample consisted of respondents ages 50 and over who answered questions on HCBS utilization and activity engagement (n = 567). Genetic matching and propensity score weighting were used to mimic randomized control and treatment groups, in order to estimate the population average treatment effect on the treated (PATT). HCBS utilization was found to be significantly associated with social engagement (PATT = 0.17, SE = 0.05, p < 0.05) and physical (PATT = -0.20, SE = 0.07, p < 0.05) engagement but not significantly associated with cognitive engagement (PATT = -0.04, SE = 0.12, p > 0.05). Sensitivity analyses found that the results were robust to the estimation model. These findings suggest that HCBS utilization is a promising model for increased activity engagement, and future policies aimed at targeting these outcomes are warranted.

